# Protonated Ethylene Carbonate: A Highly Resonance‐Stabilized Cation

**DOI:** 10.1002/open.202100229

**Published:** 2021-11-17

**Authors:** Stefanie Beck, Christoph Jessen, Andreas J. Kornath

**Affiliations:** ^1^ Department of Chemistry Ludwig-Maximilians-Universität München Butenandstr. 5–13 81377 München Germany

## Abstract

Salts containing the monoprotonated ethylene carbonate species of were obtained by reacting it with the superacidic systems *X*F/*M*F_5_ (*X*=H, D; *M*=Sb, As). The salts in terms of [C_3_H_5_O_3_]^+^[SbF_6_]^−^, [C_3_H_5_O_3_]^+^[AsF_6_]^−^ and [C_3_H_4_DO_3_]^+^[AsF_6_]^−^ were characterized by low‐temperature infrared and Raman spectroscopy. In order to generate the diprotonated species of ethylene carbonate, an excess of Lewis acid was used. However, this only led to the formation of [C_3_H_5_O_3_]^+^[Sb_2_F_11_]^−^, which was characterized by a single‐crystal X‐ray structure analysis. Quantum chemical calculations on the B3LYP/aug‐cc‐PVTZ level of theory were carried out for the [C_3_H_5_O_3_]^+^ cation and the results were compared with the experimental data. A Natural Bond Orbital (NBO) analysis revealed sp^2^ hybridization of each atom belonging to the CO_3_ moiety, thus containing a remarkably delocalized 6π‐electron system. The delocalization is confirmed by a ^13^C NMR‐spectroscopic study of [C_3_H_5_O_3_]^+^[SbF_6_]^−^.

## Graphical abstract

**The protonation of ethylene carbonate** succeeded in the superacidic media HF/*M*F_5_ (*M*=Sb, As). Surprisingly, the cation displays a highly 6π‐electron resonance stabilized CO_3_ moiety.

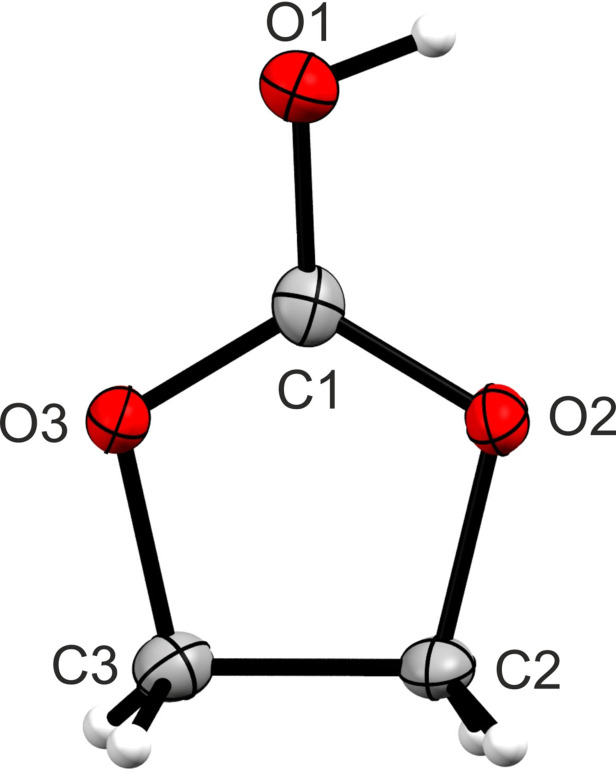

## Introduction

Lithium ion batteries play an important role in our everyday life. They are applied in mobile phones, notebooks or other battery‐operated tools.^[1]^ A lithium ion battery consists of two electrodes, which are typically separated by a semipermeable membrane, or similar separators, immersed in an ion‐conducting electrolyte. Throughout a charge process, or discharge process respectively, lithium ions are transported by an electrolyte from one electrode to the other and are intercalated in the respective layers.[^1^, ^2^, ^3^, ^4^, ^5^, ^6^] Cathode materials are commonly transition metal oxides in the form of LiMO_2_ (M=Co, Ni, Mn, Fe) or LiMn_2_O_4_ (spinel type), whereas the anode consists of carbonic materials. The electrolyte is a mixture of organic solvents, such as propylene carbonate, wherein Li salts are dissolved.^[7]^ Graphite was one of the first anode materials, which was not only able to intercalate the Li^+^ ions but also solvent molecules.^[4]^ This problem of co‐intercalation made the improvement of anode materials a greater challenge. Replacing graphite by petroleum coke prevented the solvent intercalation. Interestingly, adding ethylene carbonate to the solvent also blocked co‐intercalation into the anode material for both graphite and petroleum coke.[^4^, ^8^] This fact led to the establishment of ethylene carbonate, together with a dialkyl carbonate, as a useful electrolyte.^[1]^ Hence, many investigations on ethylene carbonate were performed, for example on the transportability of binary ethylene carbonate/ propylene carbonate systems[^7^, ^9^] or cation‐solvent interactions.^[10]^ So far, very little is known about the base properties of ethylene carbonate in literature. The only evidence for a protonated species of ethylene carbonate is given by ^1^H NMR[^11^, ^12^] and mass spectroscopic studies.^[13]^ This prompted us to study ethylene carbonate in superacidic solutions.

## Results and Discussion

### Synthesis of [C_3_H_4_ *X*O_3_]^+^[AsF_6_]^−^ and [C_3_H_5_O_3_]^+^[SbF_6_]^−^

**Scheme 1:**
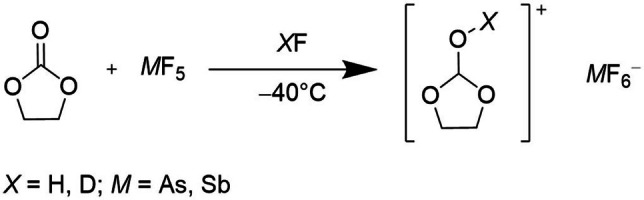
Reaction of ethylene carbonate with *M*F_5_ (*M*=As, Sb) in superacidic solutions HF/*M*F_5_.

Ethylene carbonate reacts in the superacidic solutions HF/*M*F_5_ according to Scheme 1.

The superacidic solutions were prepared using an excess of anhydrous hydrogen or deuterium fluoride, which serves as a reagent as well as a solvent. In order to obtain a complete solvation of the Lewis acid, the mixture was homogenized at −40 °C. Afterwards, ethylene carbonate was added to the frozen mixture under nitrogen atmosphere. The reaction mixture was allowed to warm up to −40 °C and salts of monoprotonated ethylene carbonate were formed. The excess of the solvent was removed in a dynamic vacuum at −78 °C overnight. The air‐ and temperature‐sensitive compounds [C_3_H_5_O_3_]^+^[SbF_6_]^−^ (**1**), [C_3_H_5_O_3_]^+^[AsF_6_]^−^ (**2**) and [C_3_H_4_DO_3_]^+^[AsF_6_]^−^ (**3**) were obtained as colorless salts. Salts **2** and **3** are stable up to room temperature, whereas **1** (containing the SbF_6_^−^ anion) decomposes at −20 °C. In consideration of the fact that ethylene carbonate bears three potential basic centers, a larger amount of Lewis acid (SbF_5_) was used in order to obtain a diprotonated compound. The excess of SbF_5_ only leads to the formation of [C_3_H_5_O_3_]^+^[Sb_2_F_11_]^−^ (**4**).

### Vibrational Spectra of [C_3_H_4_ *X*O_3_]^+^[*M*F_6_]^−^ (*M*=As, Sb and *X*=H, D)

**Figure 1:**
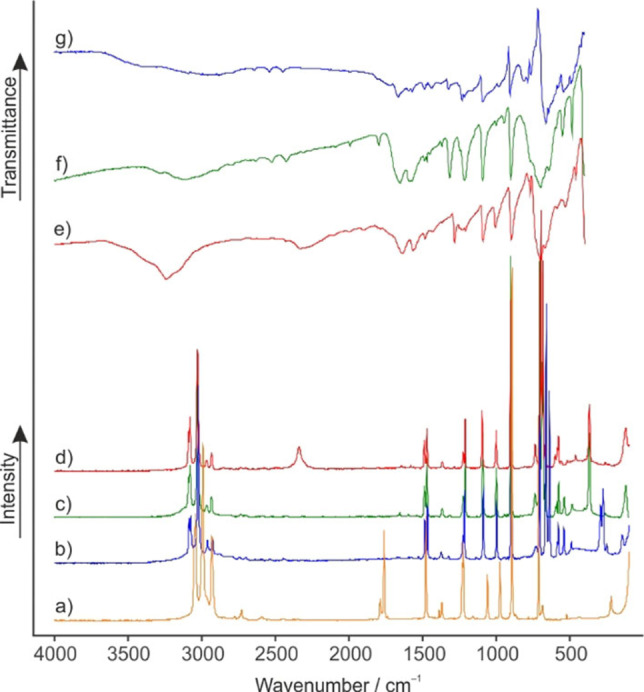
Raman spectra (a) of ethylene carbonate, (b) of [C_3_H_5_O_3_]^+^[SbF_6_]^−^ (**1**), (c) of [C_3_H_5_O_3_]^+^[AsF_6_]^−^ (**2**), and (d) of [C_3_H_4_DO_3_]^+^[AsF_6_]^−^ (**3**); IR spectra (e) of [C_3_H_4_DO_3_]^+^[AsF_6_]^−^ (**3**), (f) of [C_3_H_5_O_3_]^+^[AsF_6_]^−^ (**2**), and (g) of [C_3_H_5_O_3_]^+^[SbF_6_]^−^ (**1**).

The infrared and Raman spectra of [C_3_H_5_O_3_]^+^[SbF_6_]^−^ (**1**), [C_3_H_5_O_3_]^+^[AsF_6_]^−^ (**2**) and [C_3_H_4_DO_3_]^+^[AsF_6_]^−^ (**3**) are shown in Figure 1. Selected observed frequencies of **1**, **2** and **3** are summarized together with quantum‐chemically calculated frequencies of the free cations [C_3_H_5_O_3_]^+^ and [C_3_H_4_DO_3_]^+^ in Table 1. All observed and calculated frequencies are listed in Table S1 (see Supporting Information).

**Table 1:** Selected experimental vibrational frequencies [cm^−1^] of **1–3** and calculated vibrational frequencies [cm^−1^] of [C_3_H_5_O_3_]^+^ and [C_3_H_4_DO_3_]^+^.

[C_3_H_5_O_3_]^+^[SbF_6_]^−^ (**1**)		[C_3_H_5_O_3_]^+^[AsF_6_]^−^ (**2**)		[C_3_H_4_DO_3_]^+^[AsF_6_]^−^ (**3**)		[C_3_H_5_O_3_]^+^	[C_3_H_4_DO_3_]^+^	Assignment^[b]^
IR	Raman	IR	Raman	IR	Raman	Calc.^[a]^ (IR/Raman)	Calc.^[a]^ (IR/Raman)	
3405(vw, br)		3278(w, br)		2325 (vw, br)	2339 (9)	3694 (305/63)	2690 (176/30)	ν(O*X*)
1663 (w)	1674 (0.9)	1653 (s)	1656 (2)	1640 (w)	1646 (0.8)	1680 (461/0.8)	1667 (559/0.6)	ν_as_(CO_3_)
1591 (vw)		1587 (m)	1597 (0.7)	1567 (w)	1581 (1)	1597 (323/0.5)	1587 (253/0.6)	ν_as_(CO_3_)
1213 (w)	1218 (27)	1216 (s)	1212 (23)	826 (vw, sh)		1174 (182/1)	866 (95/0.3)	δ(CO*X*)
1086 (w)	1090 (26)	1092 (s)	1094 (32)	1092 (m)	1098 (23)	1104 (68/7)	1103 (43/6)	ν(CO)
	1000 (21)	1001 (vw)	1002 (19)	996 (w, sh)	1002 (15)	993 (2/4)	993 (18/3)	ν(CC)
902 (m)	899 (100)	901 (s)	904 (100)	900 (m)	905 (66)	897 (86/14)	899 (50/14)	skeletal breathing
784 (m)		771 (s, sh)	778 (0.5)	771 (w)		775 (28/0.006)	775 (30/0.01)	γ(CO_3_)
731 (w)	735 (3)		740 (5)	735 (s, sh)	739 (8)	743 (10/3)	740 (14/3)	δ(COC)
704 (w, sh)	707 (54)		706 (99)		710 (3)	700 (12/8)	689 (6/8)	δ(OCO)

[a] Calculated on the B3LYP/aug‐cc‐pVTZ level of theory. IR intensity in km mol^−1^ and Raman intensity in Å^4^ u^−1^. Abbreviations for IR intensities: v=very, s=strong, m=medium, w=weak. Experimental Raman activities are stated to a scale of 1 to 100. [b] *X*=H, D.

For the free cations [C_3_H_5_O_3_]^+^ and [C_3_H_4_DO_3_]^+^ with *C*_s_ symmetry, 27 fundamental vibrations are expected, active in both Raman and infrared spectra.

Due to the poor polarizability of the OH stretching vibration, the corresponding Raman lines usually are of low Raman intensity ((b) and (c)). Contrariwise, the OD stretching vibration in (d) is detected at 2339 cm^−1^. The IR spectra (f) and (g) display a broad ν(OH) band at 3406 cm^−1^ and 3278 cm^−1^_,_ respectively. In comparison, the corresponding ν(OD) of (e) is observed at 2325 cm^−1^. The red shift is in good agreement with the Teller‐Redlich rule for an H/D isotopic effect.^[14]^ These are the most meaningful vibrational modes for providing evidence for a successful protonation. Due to the *O*‐protonation, the CO double bond of ethylene carbonate is weakened, whereas the CO single bonds are strengthened. The stretching vibrations of the CO_3_ group display this tendency. Compared to the starting material, the vibration of the former CO double bond is red‐shifted by about 100 cm^−1^. In contrast, the former CO single bonds are blue‐shifted by up to 400 cm^−1^.^[15]^ The CO*X* deformation vibration is observed between 1212 cm^−1^ (c) and 1218 cm^−1^ (b) for the protonated and at 826 cm^−1^ (e) for the deuterated species. The most intense line in the Raman spectra (b–d) occurs at about 900 cm^−1^ and is assigned to the skeletal breathing mode. In comparison to ethylene carbonate (a), this mode is nearly unaffected by the protonation.[^15^, ^16^] This also applies to the rest of the skeletal vibration modes (except for the CO_3_ moiety), such as ν(CO), ν(CC), δ(COC), and δ(OCO), respectively. For the anions (*M*=Sb, As) with an ideal *O*_h_ symmetry, three Raman lines and two IR bands are expected. In the Raman spectra (b–d), more than three lines are observed and likewise the IR spectra (d–f) show more than two bands for the anions. The increased numbers of vibrations indicate a lowered symmetry of the hexafluoridometalate anions.

### Crystal Structure of [C_3_H_5_O_3_]^+^[Sb_2_F_11_]^−^ (4)

**Figure 2:**
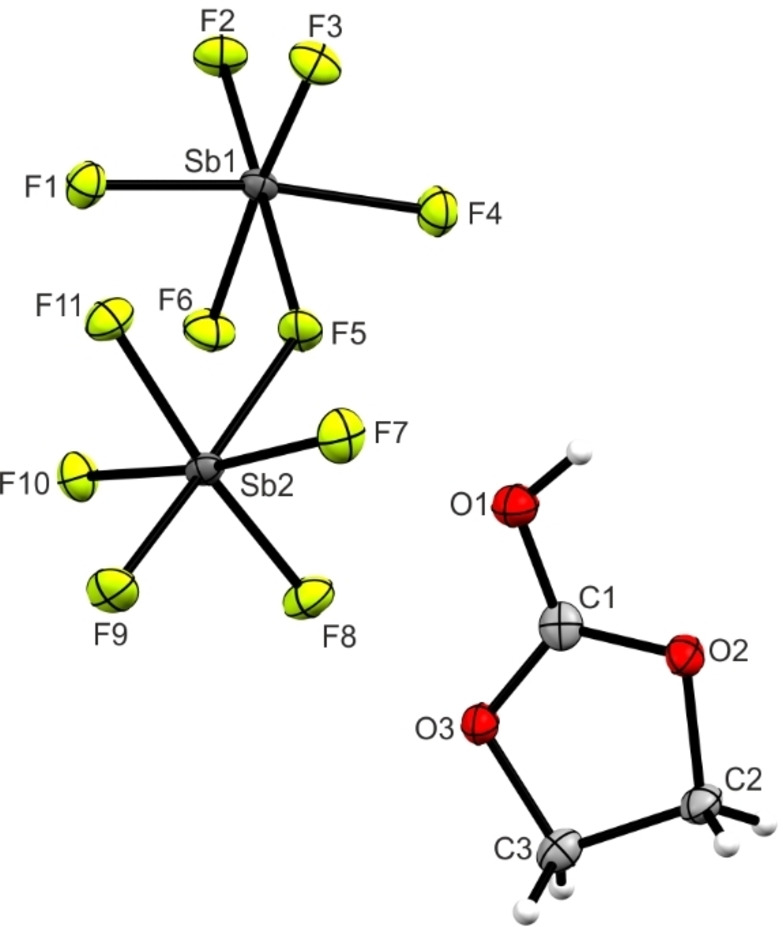
Asymmetric unit of [C_3_H_5_O_3_]^+^[Sb_2_F_11_]^−^ (**4**) (50 % probability displacement ellipsoids).

[C_3_H_5_O_3_]^+^[Sb_2_F_11_]^−^ (**4**) crystallizes in the monoclinic space group *P* 2_1_/*n* with four formula units per unit cell. The asymmetric unit is illustrated in Figure 2. Selected bond lengths and angles are summarized together with crystal structure of ethylene carbonate in Table 2.^[17^

**Table 2:** Comparison of selected bond lengths [Å] and angles [°] of ethylene carbonate and (**4**) with estimated standard deviations in parentheses. For (**4**), interatomic contacts are listed. Symmetry codes: *i*=1/2 +x, 1/2 −y,−1/2 +z; *iii*=1/2 −x, 1/2 +y, 1/2 −z.

	ethylene carbonate^[17]^	(**4**)
Bond lengths [Å]		
C1−O1	1.188(3)	1.266(3)
C1−O2	1.328(2)	1.280(4)
C1−O3	1.328(2)	1.283(3)
C2−O2	1.474(3)	1.474(3)
C3−O3	1.474(3)	1.471(3)
C3−C2	1.482(3)	1.526(4)
Bond angles [°]		
O1−C1−O3	124.2(1)	118.5(2)
O1−C1−O2	124.2(1)	123.4(3)
O3−C1−O2	111.6(2)	118.1(2)
C1−O3−C3	109.0(2)	107.8(2)
O3−C3−C2	103.2(1)	103.1(2)
C3−C2−O2	103.2(1)	103.3(2)
C2−O2−C1	109.0(2)	107.7(2)
Dihedral angles [°]		
O2−O3−C1−O1	180.00	178.5(4)
O1−C1−O2−C2	173.17	176.8(3)
O1−C1−O3−C3	173.17	175.6(2)
Interatomic contacts D‐A [Å]		
O1−H1⋅⋅⋅F11*iii*		2.663(3)
C1−F9*i*		2.753(3)

**Figure 3:**
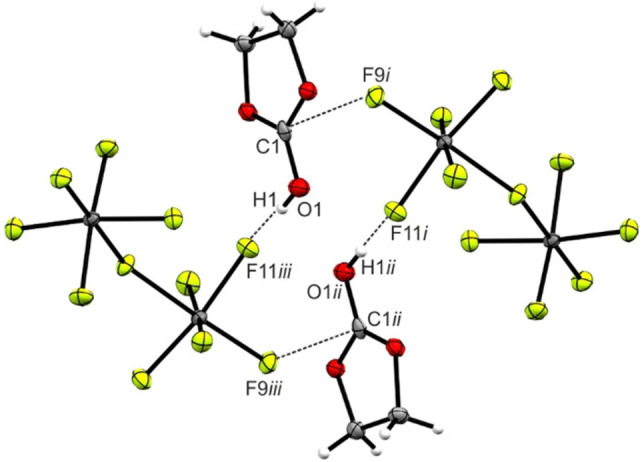
Projection of the interatomic contacts in the [C_3_H_5_O_3_]^+^[Sb_2_F_11_]^−^ (**4**) crystal. (50 % probability displacement ellipsoids). Symmetry codes: *i*=1/2 +x, 1/2 −y, −1/2 +z; *ii*=1−x, 1−y, −z; *iii*=1/2 −x, 1/2 +y, 1/2 −z.

Due to the protonation, the C1−O1 bond length is significantly elongated (1.266(3) Å) compared to the neutral compound.^[17]^ In contrast, the bond lengths from C1 to the ring oxygens (C1−O2 and C1−O3) are significantly shortened (1.280(4) Å and 1.283(3) Å). All these bond lengths are in a range between a formal single (1.43 Å) and a double bond (1.19 Å)^[18]^ and are not significantly different. The recently reported crystal structure of ethylene carbonate shows *C*_2_ symmetry with CH_2_ groups deviating out of plane. Interestingly, the CO_3_ moiety is absolutely planar.^[17]^ In consequence of the protonation, the symmetry changes to approximately *C*_s_ symmetry with dihedral angles of 175.6(2)° (O1−C1−O3−C3) and 176.8(3)° (O1−C1−O2−C2). The planarity of the CO_3_ group remains nearly unaffected (178.5(4)°). Moreover, the angles of the CO_3_ moiety approximate 120° with values in the range between 118.1(2)° (O3−C1−O2) and 123.4(3)°(O1−C1−O2). By comparing the C(H_2_)−O bond lengths (C2−O2 and C3−O3) with the neutral species, an elongation to 1.471(3) Å and 1.474(3) Å, respectively, is observed. The C−C bond length is also elongated compared with the neutral compound.^[17]^ In Figure 3, hydrogen bonds and interatomic contacts are illustrated. Cation and anion are connected by moderate hydrogen bonds O1−H1⋅⋅⋅F11*iii* (2.663(3) Å).^[19]^ Moreover, a C⋅⋅⋅F contact between C1 and F9, with a value of 2.753(3) Å below the sum of the van der Waals radii, is observed.^[20]^

The Sb−F bond lengths of the Sb_2_F_11_^−^ anion are all in a range between 1.847(2) Å and 1.887(2) Å, except the bridging Sb−F bonds. These distances are significantly longer (2.026(1) Å to 2.051(1) Å). With a Sb1−F5−Sb2 bond angle of 140.7(1)°, the Sb_2_F_11_^−^ anion possesses an angulated conformation. All these bond lengths, as well as the angles are in accordance with previously reported Sb_2_F_11_^−^ anions.[^21^, ^22^]

### Theoretical Calculations

**Figure 4:**
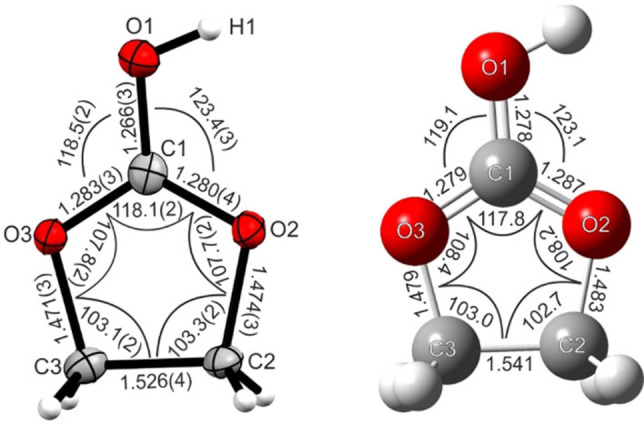
Geometric parameters of the cation of the single crystal X‐ray analysis of **4** (left) and the calculated structure of [C_3_H_5_O_3_]^+^. Bond lengths are given in Å and angles in °.

Structure optimization of the free [C_3_H_5_O_3_]^+^ cation was carried out on the B3LYP/aug‐cc‐pVTZ level of theory. IR and Raman intensities as well as vibrational frequencies were calculated in the harmonic approximation. Figure 4 shows the comparison of the cation of the single‐crystal X‐ray structure (4) and the calculated structure of [C_3_H_5_O_3_]^+^ together with bond lengths and angles.

**Scheme 2:**
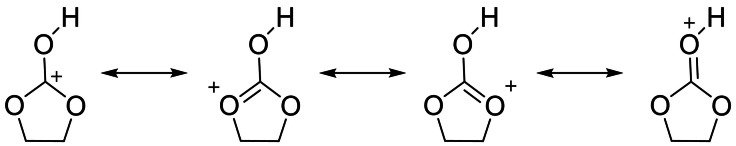
Lewis resonance structures of monoprotonated ethylene carbonate.

Comparing the values of the experimentally obtained geometric parameters with those obtained from the calculation shows that all values are in good agreement. Only the C1−O1 bond length is overestimated by the calculation. The crystal structure analysis as well as the quantum chemical calculation indicate that all CO bond lengths of the CO_3_ moiety are of approximately equal length. This is caused by the resonance stabilization of this group and can be expressed by the Lewis resonance structures shown in Scheme 2.

**Figure 5:**
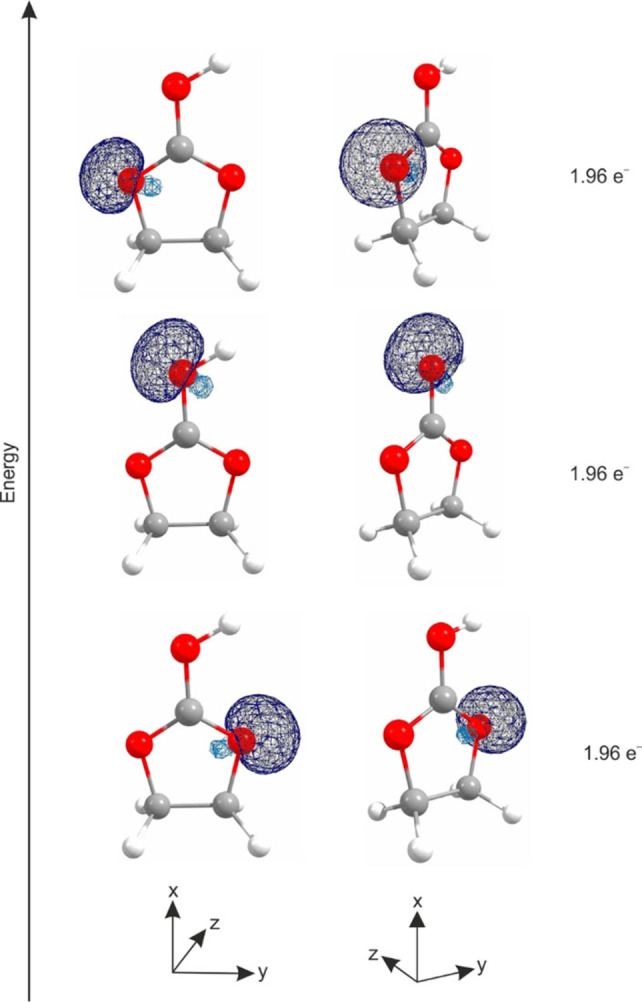
Calculated NBOs (view: frontal and perspective) of the oxygen atoms; here: lone pairs, together with electron occupancy.

Despite the protonation, the sp^2^ hybridization of the central carbon atom is conserved compared to the neutral compound. Even more interesting is the hybridization of the oxygen atoms. In order to investigate the hybridization situation of the CO_3_ group, a Natural Bond Orbital (NBO) analysis was performed on the B3LYP/aug‐cc‐pVTZ level of theory. In Figure 5, the calculated NBOs of the lone pairs on the oxygen atoms together with the calculated electron occupancy are illustrated.

**Figure 6:**
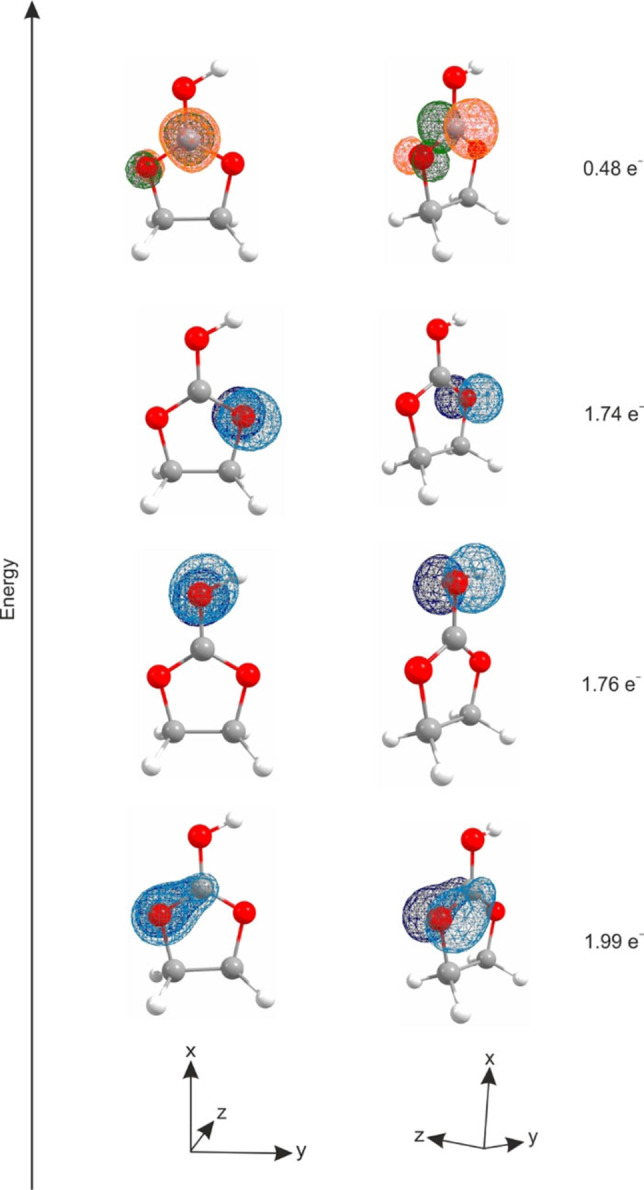
Calculated NBOs (view frontal and perspective) of oxygen atoms and the central carbon atom; here: p‐orbitals, together with calculated electron occupancy.

All oxygen lone pairs are located in the molecule plane, which suggests sp^2^ hybridization on each oxygen atom in the protonated species. In order to confirm this hybridization, the corresponding p‐orbitals were considered. Figure 6 shows the calculated NBOs of the corresponding p‐orbitals together with the calculated electron occupancy. The p‐orbital of the central carbon atom as well as every p‐orbital of the oxygen atoms are oriented perpendicular to the molecule plane. The NBO analysis shows a π‐bond between O3 and C1 with approximately two electrons, whereas the residual p‐orbitals on O2 and O1 are occupied with 1.74 and 1.76 electrons, respectively. Additionally, the NBO of the antibonding π‐bond of O3 and C1 is occupied with 0.48 electrons. In Table S3, selected NBOs (concerning the CO_3_ group) are listed, together with calculated values for electron occupancy and s‐ and p‐character given in percentage (see Supporting Information). In summary, the NBO analysis indicates that a strong delocalization of the electrons over the Y‐shaped CO_3_ group is possible.

### NMR Spectroscopy of [C_3_H_5_O_3_]^+^[SbF_6_]^−^ (1)

The NMR spectroscopic study of [C_3_H_5_O_3_]^+^[SbF_6_]^−^ (**1**) was carried out in anhydrous HF (*a*HF). For better comparability, a reference of neutral ethylene carbonate was measured in *a*HF as well. The chemical shifts obtained by ^1^H, ^13^C and ^19^F NMR spectroscopy are listed in Table 3.

**Table 3:** ^1^H, ^13^C and ^19^F chemical shifts of ethylene carbonate and [C_3_H_5_O_3_]^+^[SbF_6_]^−^ (**1**) recorded in *a*HF at room temperature for the neutral compound and at −40 °C for **1**, respectively.

	ethylene carbonate	[C_3_H_5_O_3_]^+^[SbF_6_]^−^ (**1**)
δ^1^H [ppm]	4.54 (CH_2_)	4.75 (CH_2_)
	7.80 (HF)	8.04 (HF)
δ ^13^C [ppm]	69.73 (CH_2_)	73.11 (CH_2_)
	165.58 (CO_3_)	168.16 (CO_3_)
δ ^19^F [ppm]	−197.97 (HF)	−196.41 (HF)
		−123.24 (SbF_6_^−^)

Due to the large excess of *a*HF, the resonance of the proton on the oxygen atom is not observable. Nevertheless, the observation of the anion confirms the formation of the [C_3_H_5_O_3_]^+^ cation. The ^19^F signal at −123.24 ppm is attributed to the SbF_6_^−^ anion, which is in accordance with literature.^[23]^ In the ^1^H NMR spectrum, the signal of the protons of the CH_2_ groups, which appears as a multiplet, is slightly shifted to higher frequencies compared to ethylene carbonate. The same trend is observed for the corresponding C signal in the^13^C NMR spectrum. Interestingly, the C atom, belonging to the CO_3_ group, is also only slightly deshielded. The signal is shifted by about 2.58 ppm. This leads to the conclusion that protonated ethylene carbonate is a cation where the positive charge is strongly delocalized over the CO_3_ moiety.

## Conclusion

The protonation of ethylene carbonate succeeded for the first time in the superacidic systems HF/*M*F_5_ (*M*=As, Sb) and the salts [C_3_H_5_O_3_]^+^[SbF_6_]^−^ (**1**), [C_3_H_5_O_3_]^+^[AsF_6_]^−^ (**2**), [C_3_H_4_DO_3_]^+^[AsF_6_]^−^ (**3**) and [C_3_H_5_O_3_]^+^[Sb_2_F_11_]^−^ (**4**) were isolated. The compounds were characterized by IR and Raman spectroscopy and, in the case of **4**, by an X‐ray structure analysis. For compound **1**, an NMR‐spectroscopic study in *a*HF was carried out. The experimental results were compared with quantum chemical calculations on the B3LYP/aug‐cc‐pVTZ level of theory. To elucidate the bonding situation of the CO_3_ moiety, a NBO analysis was performed. This calculation indicates a sp^2^ hybridization on the central carbon atom as well as on the oxygen atoms, thus leading to the conclusion that protonated ethylene carbonate is a compound with a remarkable 6π‐electron delocalization, located on the CO_3_ group.

## Experimental Section

### General

Caution! Avoid contact with any of these compounds. The hydrolysis of all these salts might form HF, which burns skin and causes irreparable damage. Safety arrangements should be taken while using and handling these materials.

All reactions were performed by standard Schlenk technique using a stainless steel vacuum line. All reactions in superacidic media were carried out in FEP/PFA reactors closed with a stainless steel valves. The vacuum line as well as the reactors were dried with fluorine prior to use. Detailed Information about the used apparatus and materials as well as analytic measurement methods are described in the Supporting Information.

Deposition Number 1978961 (for **4**) contains the supplementary crystallographic data for this paper. These data are provided free of charge by the joint Cambridge Crystallographic Data Centre and Fachinformationszentrum Karlsruhe Access Structures service.

### Synthesis of [C_3_H_5_O_3_]^+^[SbF_6_]^−^ (1) and [C_3_H_5_O_3_]^+^[Sb_2_F_11_]^−^ (4)

First, antimony pentafluoride SbF_5_ (140 mg, 0.65 mmol, 1.0 eq. for **1** and 170 mg, 0.78 mmol, 2.0 eq. for **4**) was condensed into a FEP tube reactor at −196 °C. Afterwards, 2 mL anhydrous hydrogen fluoride *a*HF (2 mL) were also condensed in the reactor. To form the superacidic system, both compounds (SbF_5_ and HF) were allowed to warm up to −40 °C. The reaction mixture was cooled to −196 °C and under inert gas atmosphere ethylene carbonate was added (57 mg, 0.65 mmol, 1.0 eq. for **1** and 34 mg, 0.39 mmol, 1.0 eq. for **4**). For the desired protonation of ethylene carbonate, the reaction mixture was warmed up again to −40 °C and homogenized until the salt was completely dissolved. To obtain salt **1**, the solution was again cooled (−196 °C) and excess HF was removed overnight at −78 °C in a dynamic vacuum. For the crystallization of compound **4**, the reactor was left in an ethanol bath (T=−50 °C) until the salt recrystallized. Both salts [C_3_H_5_O_3_]^+^[SbF_6_]^−^ (**1**) and [C_3_H_5_O_3_]^+^[Sb_2_F_11_]^−^ (**4**) were obtained as colorless solids, which are stable under inert gas atmosphere up to −20 °C.

### Synthesis of [C_3_H_5_O_3_]^+^[AsF_6_]^−^ (2) and [C_3_H_4_DO_3_]^+^[AsF_6_]^−^ (3)

Anhydrous hydrogen fluoride *a*HF (2 mL) for **2**, respectively deuterium fluoride *a*DF for **3**, was condensed into a FEP tube‐reactor at −196 °C. Under the same conditions, arsenic pentafluoride (85 mg, 0.5 mmol, 1.0 eq.) was also condensed into the reactor. Both compounds were warmed up to −40 °C, blended to form the superacidic system and cooled to −196 °C. Ethylene carbonate (44 mg, 0.5 mmol, 1.0 eq.) was added under inert gas atmosphere and the reaction mixture was again allowed to warm up to −40 °C. After homogenizing all compounds until the salt was completely dissolved, the solution was again cooled to −196 °C and excess *a*HF/*a*DF was removed in a dynamic vacuum overnight at −78 °C. [C_3_H_5_O_3_]^+^[AsF_6_]^−^ (**2**) and [C_3_H_4_DO_3_]^+^[AsF_6_]^−^ (**3**) were obtained as colorless solids, which are stable up to room temperature.

## Conflict of interest

The authors declare no conflict of interest.

## Supporting information

As a service to our authors and readers, this journal provides supporting information supplied by the authors. Such materials are peer reviewed and may be re‐organized for online delivery, but are not copy‐edited or typeset. Technical support issues arising from supporting information (other than missing files) should be addressed to the authors.

Supporting Information
