# Supplementary material for: Protonated Ethylene Carbonate: A Highly Resonance‐Stabilized Cation
Source: ChemistryOpen. 2021 Nov 17;10(12):1160–5. doi: 10.1002/open.202100229 (PMC8634765; doi:10.1002/open.202100229)
Supplement: Supplementary file 1 — Supporting Information [file OPEN-10-1160-s001.pdf]

ChemistryOpen

Supporting Information

Protonated Ethylene Carbonate: A Highly Resonance-Stabilized Cation

Stefanie Beck, Christoph Jessen, and Andreas J. Kornath*

Table of Contents

Apparatus and Materials

Table S1. Experimental vibrational frequencies [cm^{-1}] of **1**-**3** and calculated vibrational frequencies [cm^{-1}] of free cations $[C_3H_5O_3]^+$ and $[C_3H_4DO_3]^+$.

Table S2. Crystal data structure refinement for $[C_3H_5O_3]^+[Sb_2F_{11}]^-$ (**4**).

Table S3. Selected NBOs (BD = 2-center bond; LP = 1-center valence lone pair; BD* = 2-center antibond) together with calculated values for occupancy and s- and p-character.

Table S4. Cartesian coordinates of calculated minimum structures of protonated ethylene carbonate at the B3LYP/aug-cc-pVTZ level of theory.

Figure S1. 400 MHz ^{1}H NMR spectrum of ethylene carbonate.

Figure S2. 100 MHz ^{13}C NMR spectrum of ethylene carbonate.

Figure S3. 376 MHz ^{19}F NMR spectrum of ethylene carbonate.

Figure S4. 400 MHz ^{1}H NMR Spectrum of $[C_3H_5O_3]^+[SbF_6]^-$ (**1**).

Figure S5. 100 MHz ^{13}C NMR spectrum of $[C_3H_5O_3]^+[SbF_6]^-$ (**1**).

Figure S6. 376 MHz ^{19}F Spectrum of $[C_3H_5O_3]^+[SbF_6]^-$ (**1**).

Apparatus and Materials

Low-temperature IR spectra were recorded by a Bruker Vertex-80V FTIR spectrometer ($\tilde{\nu}$ = 350 to 4000 cm^{-1}). For the measurement, small amounts of the synthesized compounds were placed on a CsBr single-crystal plate in a cooled cell.

Raman measurements were carried out on a Bruker MultiRAM FT-Raman spectrometer with Nd:YAG laser excitation (λ = 1064 nm) in vacuum at –196 °C. Therefore, the samples were transferred into cooled glass cells.

The low-temperature X-ray diffraction of $[C_3H_5O_3]^+[Sb_2F_{11}]^-$ (**4**) was performed with an Oxford XCalibur3 diffractometer equipped with a Kappa CCD detector, operated with Mo-κ_α radiation (λ = 0.71073 Å) and a Spellman generator (voltage 50 kV, current 40 mA). The collection of the data was performed at 113 K using the CrysAlis CCD software,[1] while the reduction were carried out using CrysAlis RED software.[2] Programs SHELXS[3] and SHELXL-97,[4] belonging to the WinGX software package, were used for performing the solution and refinement of the crystal structure. Afterwards the structure was checked by PLATON software.[5] The absorption correction was accomplished by using the SCALE3 ABSPACK multiscan method.[6] In Table S2 selected data and parameters of the X-ray structure analysis are listed. Crystallographic data (excluding structure factors) for the structure in this paper have been deposited with the Cambridge Crystallographic Data Centre, CCDC, 12 Union Road, Cambridge CB21EZ, UK. Copies of the data can be obtained free of charge on quoting the depository number CCDC-1978961 for $[C_3H_5O_3]^+[Sb_2F_{11}]^-$ (**4**) (Fax: +44-1223-336-033; E-Mail: deposit@ccdc.cam.ac.uk, http:// www.ccdc.cam.ac.uk).

Quantum chemical calculations were carried out on the B3LYP/aug-cc-PVTZ level of theory by Gaussian 09.[7]

NMR spectra were recorded on a Jeol ECX400 NMR. The spectrometer were externally referenced to $CFCl_3$ for ^{19}F and to tetramethylsilane for ^{1}H and ^{13}C NMR spectra. The spectra were recorded inside 4 mm FEP NMR tube inliners. Acetone-d_6 was employed for external shimming, when *a*HF was used as solvent for the respective compounds. The NMR samples were prepared by dissolving ethylene carbonate, and the protonated species $[C_3H_5O_3]^+[SbF_6]^-$ (**1**) respectively, in *a*HF. The solutions were transferred into a 4 mm FEP NMR tube inliner. The inliner was than frozen and flame sealed.

[1] *CrysAlisCCD, Version 1.171.35.11 (release 16-05-2011 CrysAlis 171.NET)*, Oxford Diffraction Ltd., **2011**.
[2] *CrysAlisRED, Version 1.171.35.11 (release 16.05.2011 CrysAlis 171.NET)*, Oxford Diffraction Ltd., **2011**.
[3] G. Sheldrick, *SHELXS-97, Program for Crystal Structure Solution*, University of Göttingen, Germany, **1997**.
[4] G. Sheldrick, *SHELXL-97, Program for the Refinement of Crystal Structures*, University of Göttingen, Germany, **1997**.
[5] A. Spek, *PLATON, A Multipurpose Crystallographic Tool*, Utrecht University, Utrecht (The Netherlends), **1999**.
[6] *SCALE3 ABSPACK - An Oxford Diffraction Program*, Oxford Diffraction Ltd., UK, **2005**.
[7] M. J. Frisch, G. W. Trucks, H. B. Schlegel, G. E. Scuseria, M. A. Robb, J. R. Cheeseman, G. Scalmani, V. Barone, B. Mennucci, G. A. Petersson, H. Nakatsuji, M. Caricato, X. Li, H. P. Hratchian, A. F. Izmaylov, J. Bloino, G. Zheng, J. L. Sonnenberg, M. Hada, M. Ehara, K. Toyota, R. Fukuda, J. Hasegawa, M. Ishida, T. Nakajima, Y. Honda, O. Kitao, H. Nakai, T. Vreven, J. A. Montgomery, Jr., J. E. Peralta, F. Ogliaro, M. Bearpark, J. J. Heyd, E. Brothers, K. N. Kudin, V. N. Staroverov, R. Kobayashi, J. Normand, K. Raghavachari, A. Rendell, J. C. Burant, S. S. Iyengar, J. Tomasi, M. Cossi, N. Rega, J. M. Millam, M. Klene, J. E. Knox, J. B. Cross, V. Bakken, C. Adamo, J. Jaramillo, R. Gomperts, R. E. Stratmann, O. Yazyev, A. J. Austin, R. Cammi, C. Pomelli, J. W. Ochterski, R. L. Martin, K. Morokuma, V. G. Zakrzewski, G. A. Voth, P. Salvador, J. J. Dannenberg, S. Dapprich, A. D. Daniels, Ö. Farkas, J. B. Foresman, J. V. Ortiz, J. Cioslowski, D. J. Fox, *Gaussian 09, Revision A.02*, Gaussian Inc., Wallingford CT, **2009**.

Table S1. Experimental vibrational frequencies [cm^{-1}] of **1**-**3** and calculated vibrational frequencies [cm^{-1}] of free cations $[C_3H_5O_3]^+$ and $[C_3H_4DO_3]^+$.

**$[C_3H_5O_3]^+[SbF_6]^-$**		**$[C_3H_5O_3]^+[AsF_6]^-$**		**$[C_3H_4DO_3]^+[AsF_6]^-$**		**$[C_3H_5O_3]^+$**	**$[C_3H_4DO_3]^+$**	**Assignment[b]**
**IR**	**Raman**	**IR**	**Raman**	**IR**	**Raman**	**Calc.[a] (IR/Raman)**	**Calc.[a] (IR/Raman)**	
3405(vw, br)		3278 (vw)		2325 (vw, br)	2339 (9)	3694 (305/63)	2690 (176/30)	ν(O*X*)
3083 (vw)	3085 (5)	3108 (w)	3089 (3)	3089 (w, sh)	3090 (5)	3189 (3/42)	3189 (3/42)	$\nu_{as}(CH_2)$
	3074 (12		3078 (16)		3079 (18)	3174 (0.01/48)	3174 (0.01/48)	$\nu_{as}(CH_2)$
						3118 (0.3/52)	3118 (0.3/52)	$\nu_s(CH_2)$
	3024 (68)		3028 (54)	3031 (vw, sh)	3028 (47)	3117 (0.4/161)	3117 (0.4/161)	$\nu_s(CH_2)$
	2961 (4)		2966 (3)	2961 (vw)	2968 (3)			?
	2924 (6)		2932 (6)		2931 (6)			?
2637 (vw)		2630 (vw)						?
2534 (vw)		2522 (vw)						?
2442 (vw)	2445 (0.8)	2424 (vw)						?
1663 (w)	1674 (0.9)	1653 (s)	1656 (2)	1640 (w)	1646 (0.8)	1680 (461/0.8)	1667 (559/0.6)	$\nu_{as}(CO_3)$
1591 (vw)		1587 (m)	1597 (0.7)	1567 (w)	1581 (1)	1597 (323/0.5)	1587 (253/0.6)	$\nu_{as}(CO_3)$
	1531 (1)		1539 (0.5)			1529 (0.9/4)	1528 (1/4)	$\delta(CH_2)$
1483 (vw)	1487 (13)		1489 (8)	1483 (w)	1490 (9)			?
1468 (vw)	1470 (20)	1471 (w)	1473 (23)	1469 (vw)	1473 (16)	1514 (3/6)	1514 (1/6)	$\delta(CH_2)$
1371 (vw)	1376 (3)	1370 (w)	1369 (4)	1369 (w)	1369 (3)	1394 (1/0.7)	1394 (0.5/0.7)	$\omega(CH_2)$
1320 (vw)	1325 (1)	1316 (s)	1317 (0.8)	1284 (w)	1289 (0.7)	1330 (90/0.4)	1314 (89/0.2)	$\omega(CH_2)$
1250 (vw, sh)		1247(w, sh)				1244 (0.05/2)	1244 (0.03/2)	$\rho(CH_2)$
1227 (w)	1229 (4)		1226 (3)	1228 (w)	1227 (5)	1229 (5/3)	1229 (5/3)	$\tau(CH_2)$
1213 (w)	1218 (27)	1216 (s)	1212 (23)	826 (vw, sh)		1174 (182/1)	866 (95/0.3)	δ(CO*X*)
1164 (vw)		1133 (vw)				1145 (0.04/0.06)	1145 (0.004/0.05)	$\tau(CH_2)$
1086 (w)	1090 (26)	1092 (s)	1094 (32)	1092 (m)	1098 (23)	1104 (68/7)	1103 (43/6)	ν(CO)
	1000 (21)	1001 (vw)	1002 (19)	1007 (w)	1002 (15)	993 (2/4)	993 (18/3)	ν(CC)
977 (w)		947 (vw)		992 (w, sh)		931 (7/0.7)	991(29/1))	ν(CO)
902 (m)	899 (100)	901 (s)	904 (100)	900 (m)	905 (66)	897 (86/14)	899 (50/14)	skeletal breathing
806 (m, sh)				826 (vw, sh)		847 (0.2/0.1)	847 (0.2/0.1)	$\rho(CH_2)$
784 (m)		771 (s, sh)	778 (0.5)	771 (w)		775 (28/0.006)	775 (30/0.01)	$\gamma(CO_3)$
762 (m)								?
731 (w)	735 (3)		740 (5)	735 (s, sh)	739 (8)	743 (10/3)	740 (14/3)	δ(COC)
704 (w, sh)	707 (54)		706 (99)		710 (3)	700 (12/8)	689 (6/8)	δ(OCO)
						565 (135/0.8)	418 (63/0.2)	$\delta(COX)_{oop}$
						451 (14/0.3)	424 (15/0.3)	δ(OCO)
	253 (3)		264 (1)		263 (1)	229 (9/0.1)	226 (11/0.08)	skeletal vibration
	148 (5)		124 (12)		124 (8)	36 (0.2/0.02)	36 (0.2/0.02)	skeletal vibration

658 (vs)	662 (97)	699 (vs)	692 (28)	700 (vs)	699 (100)		$[MF_6]^-$
641 (vs)	642 (59)	676 (vs, sh)	685 (75)	672 (vs)	686 (65)		$[MF_6]^-$
621 (vs)	581 (12)	642 (vs)	671 (7)	589 (m)	672 (6)		$[MF_6]^-$
611 (vs)	543 (10)	595 (m, sh)	634 (0.5)	587 (m, sh)	601 (3)		$[MF_6]^-$
596 (vs)	493 (5)	551 (m)	590 (5)	532 (m)	587 (1)		$[MF_6]^-$
578 (vs)	294 (8)	487 (s)	579 (13)	464 (w)	580 (12)		$[MF_6]^-$
542 (m)	278 (26)		541 (7)		565 (1)		$[MF_6]^-$
491 (vs)			488 (4)		519 (1)		$[MF_6]^-$
467 (s)			401 (0.7)		464 (3)		$[MF_6]^-$
452 (s)			372 (32)		372 (24)		$[MF_6]^-$
422 (m)							$[MF_6]^-$

[a] Calculated on the B3LYP/aug-cc-pVTZ level of theory. IR intensity in km/mol and Raman intensity in $Å^4/u$. Abbreviations for IR intensities: v = very, s = strong, m = medium, w = weak. Experimental Raman activities are stated to a scale of 1 to 100. [b] *X* = H, D.

Table S2. Crystal data and structure refinement for $[C_3H_5O_3]^+[Sb_2F_{11}]^-$ (**4**).

	$[C_3H_5O_3]^+[Sb_2F_{11}]^-$ (**4**)
Empirical formula	C3 H5 F11 O3 Sb2
M_r	541.57
Crystal system	monoclinic
Space group	$P\ 2_1/n$
a [Å]	9.0783(4)
b [Å]	10.4388(4)
c [Å]	12.3161(4)
α [°]	90
β [°]	93.146(4)
γ [°]	90
V [Å^3]	1165.40(8)
Z	4
ρ_{calcd} [gcm^{-3}]	3.087
μ[mm^{-1}]	4.785
$\lambda_{Mo\kappa\alpha}$	0.71073
F(000)	992
T[K]	113(2)
hkl range	–12:12; –12:14;–17:17
refl. measured	12132
refl. unique	3542
R_{int}	0.0311
parameters	178
R(F)/*w*R(F^2)[a)]	0.0348/0.0531
weighting scheme[b)]	0.0157
S(GoF)[c)]	1.071
residual density [eÅ^{-3}]	1.159/ –0.813
devide type	Oxford XCalibur
solution/refinement	SHELXS-97
CCDC	1978961

a) $R_1 = \Sigma||F_o|-|F_c||/\Sigma|F_o|$; b) $wR_2 = [\Sigma[w(F_o^2-F_c^2)^2]/\Sigma[w(F_o)^2]]^{1/2}$; $w = [\sigma_c^2(F_o^2)+(xP)^2+yP]^{-1}$; $P = (F_o^2+2F_c^2)/3$ c) GoF = $\{\Sigma[w(F_o^2-F_c^2)^2]/(n-p)\}^{1/2}$ (n = number of reflexions; p = total number of parameters).

Table S3. Selected NBOs (BD = 2-center bond; LP = 1-center valence lone pair; BD* = 2-center antibond) together with calculated values for occupancy and s- and p-character.[a]

**Bond**	**Occupancy**	**s-, p-character**
BD(1) C1–O3	1.99	O3 s (30.19%), p 2.29 (69.24%)
		C1 s (33.29%), p 2.00 (66.57%)
BD(2) C1–O3	1.99	O3 s (0.00%), p 1.00 (99.55%)
		C1 s (0.00%), p 1.00 (99.47%)
BD(1) O2–C1	2.00	O2 s (29.69%), p 2.35 (69.76%)
		C1 s (32.95%), p 2.03 (66.91%)
BD(1) C1–O1	1.99	C1 s (33.65%), p 1.97 (66.24%)
		O1 s (32.76%), p 2.03 (66.63%)
LP(1) O3	1.96	s (44.68%), p 1.23 (55.13%)
LP(1) O2	1.96	s (44.70%), p 1.23 (55.13%)
LP(2) O2	1.74	s (0.00%), p 1.00 (99.56%)
LP(1) O1	1.96	s (44.28%), p 1.25 (55.48%)
LP(2) O1	1.76	s (0.00%), p 1.00 (99.55%)
BD*(1) O3–C1	0.05	O3 s (30.19%), p 2.29 (69.24%)
		C1 s (33.29%), p 2.00 (66.57%)
BD*(2) O3–C1	0.48	O3 s (0.00%), p 1.00 (99.55%)
		C1 s (0.00%), p1.00 (99.47%)

[a] Calculated on the B3LYP/aug-cc-pVTZ level of theory.

Table S4. Cartesian coordinates of calculated minimum structures of protonated ethylene carbonate at the B3LYP/aug-cc-pVTZ level of theory.

Atoms	x	y	z
C	-1.399518	-0.742857	-0.000086
C	-1.363250	0.798016 0	0.000090
H	-1.832686	-1.179328	0.893609
H	-1.832406	-1.179118	-0.894021
H	-1.772339	1.255483	0.894512
H	-1.772615	1.255698	-0.894092
O	0.033739	-1.108557	0.000084
O	0.090864	1.088526	-0.000089
C	0.724734	-0.031504	-0.000002
O	2.000313	-0.111526	-0.000008
H	2.438917	0.757801	0.000079

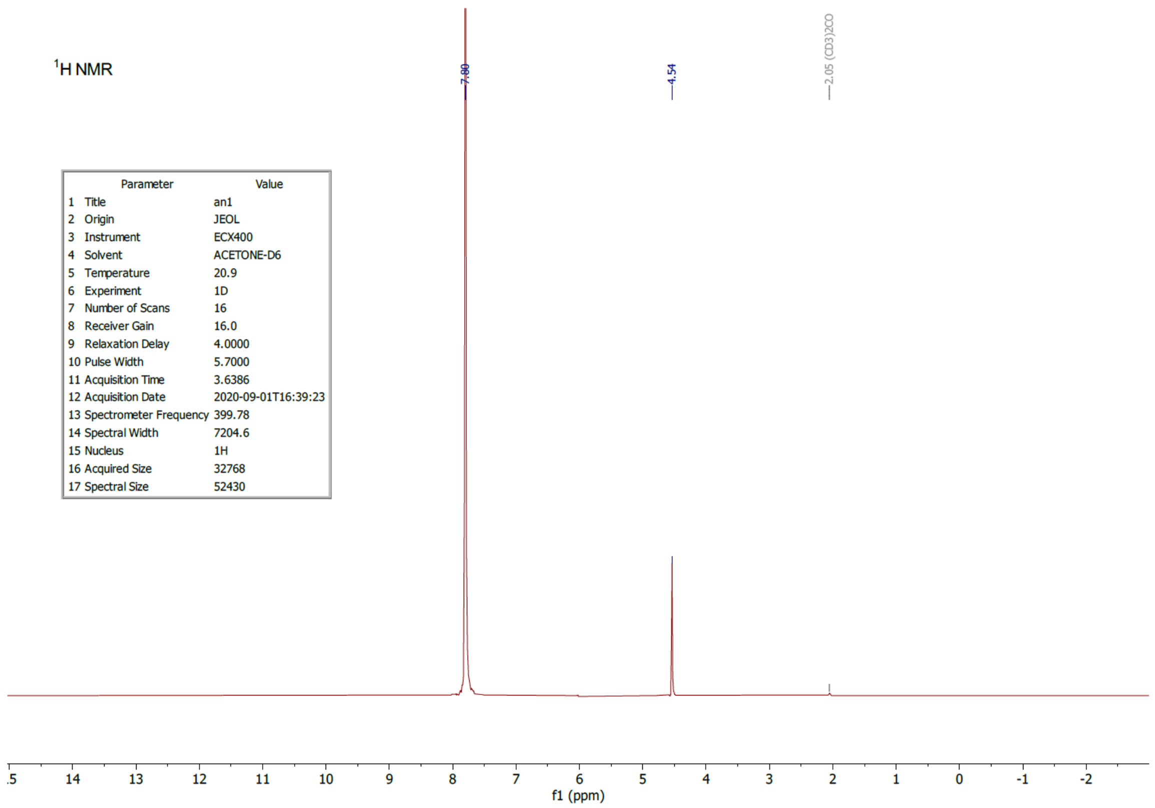

Figure S1. 400 MHz ^{1}H NMR spectrum of ethylene carbonate.

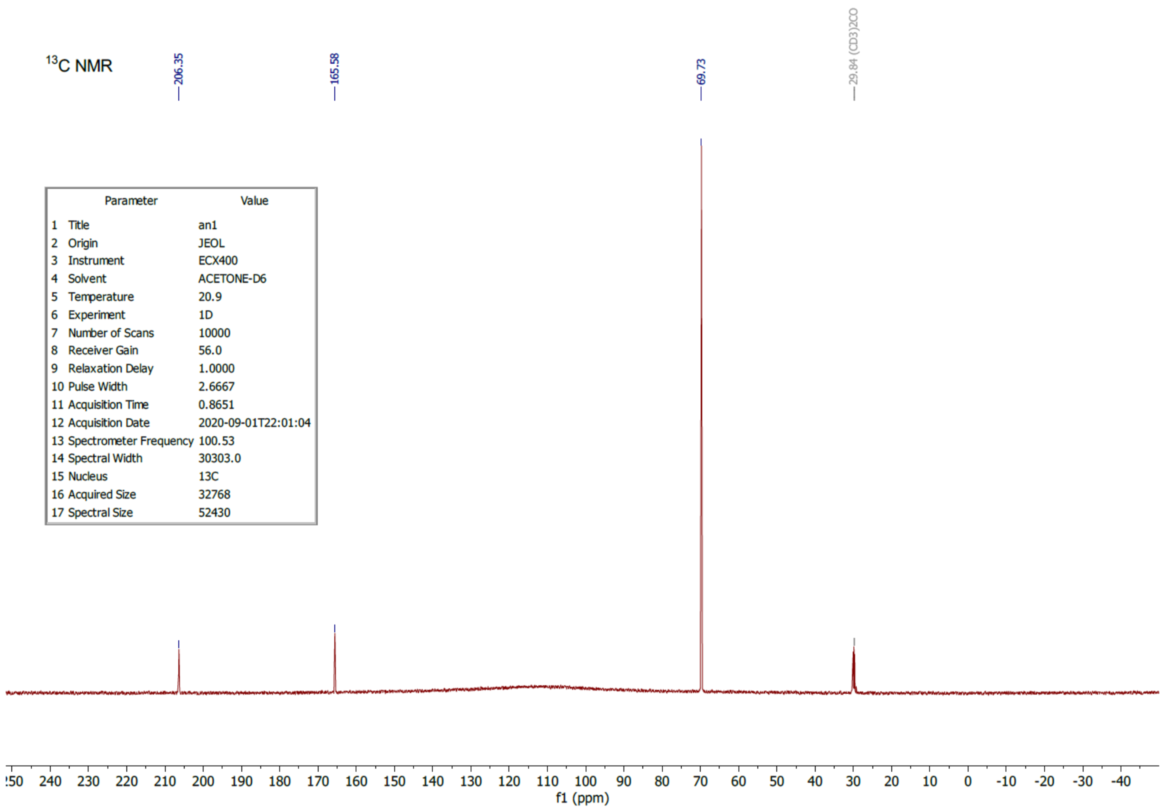

Figure S2. 100 MHz ^{13}C NMR spectrum of ethylene carbonate.

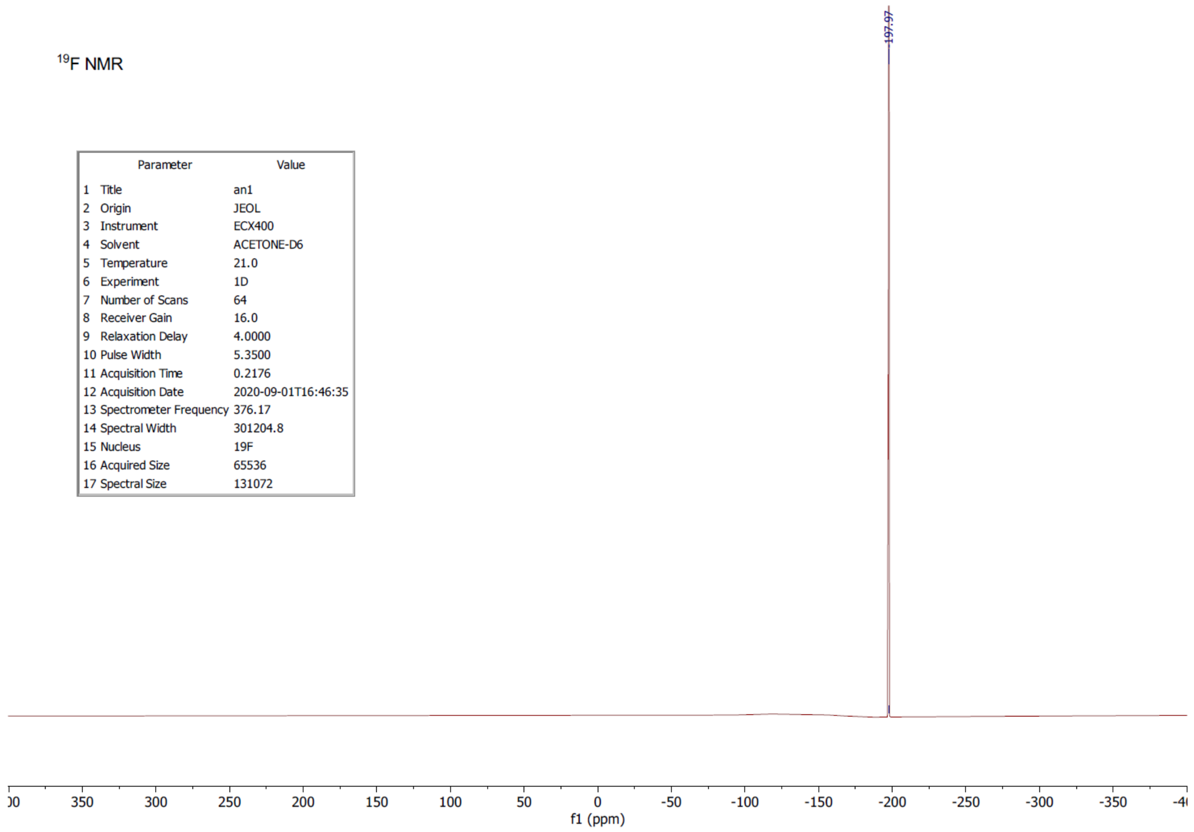

Figure S3. 376 MHz ^{19}F NMR spectrum of ethylene carbonate.

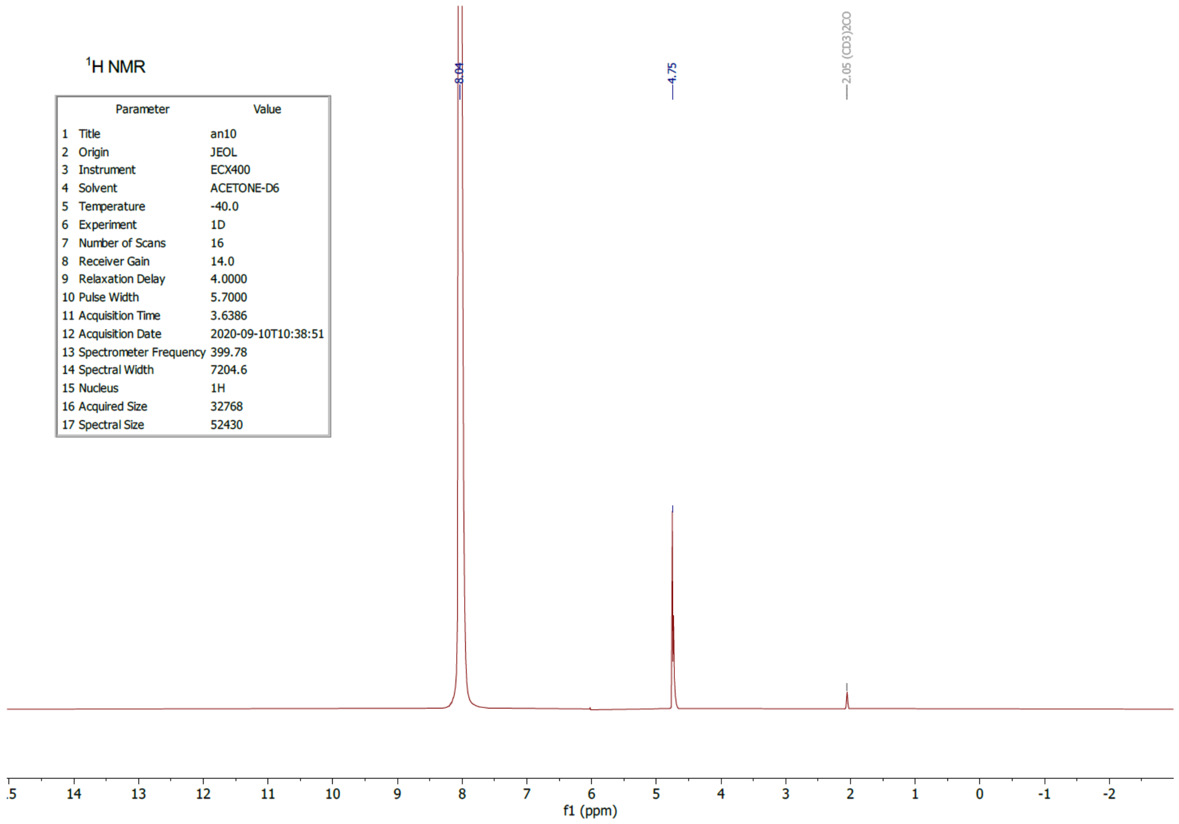

Figure S4. 400 MHz ^{1}H NMR Spectrum of $[C_3H_5O_3]^+[SbF_6]^-$ (**1**).

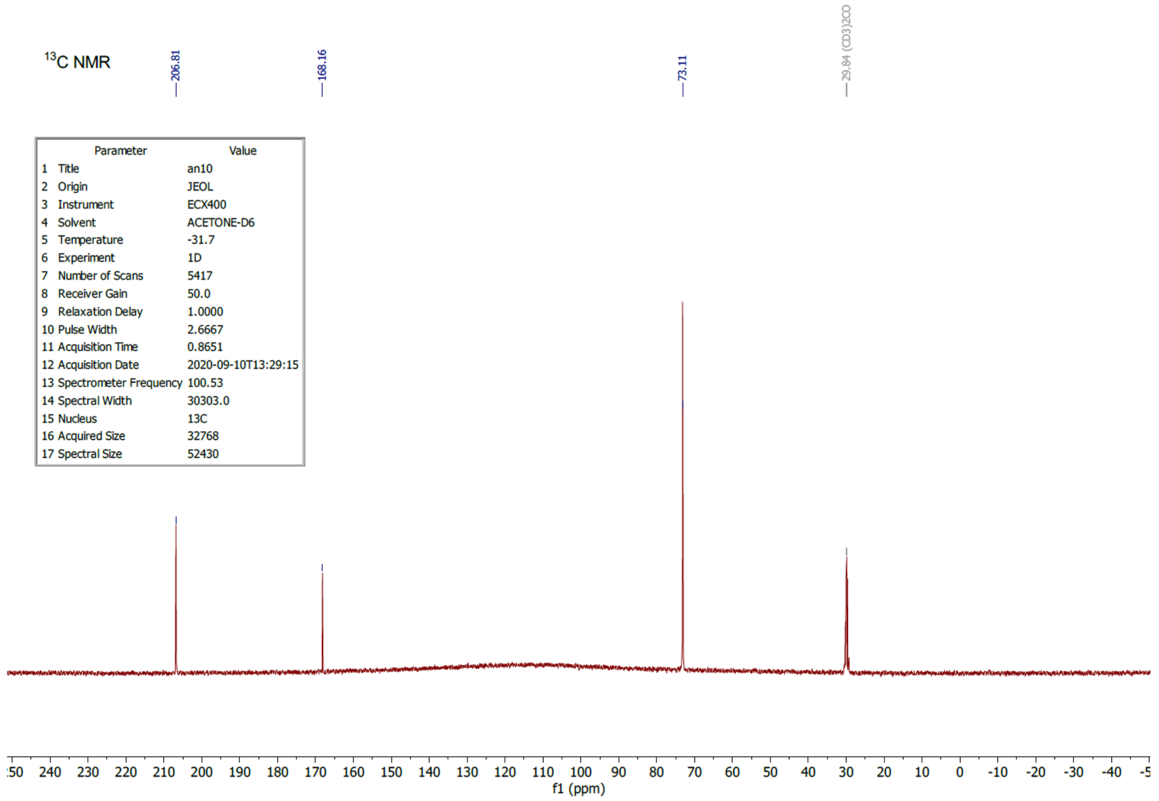

Figure S5. 100 MHz ^{13}C NMR spectrum of $[C_3H_5O_3]^+[SbF_6]^-$ (**1**).

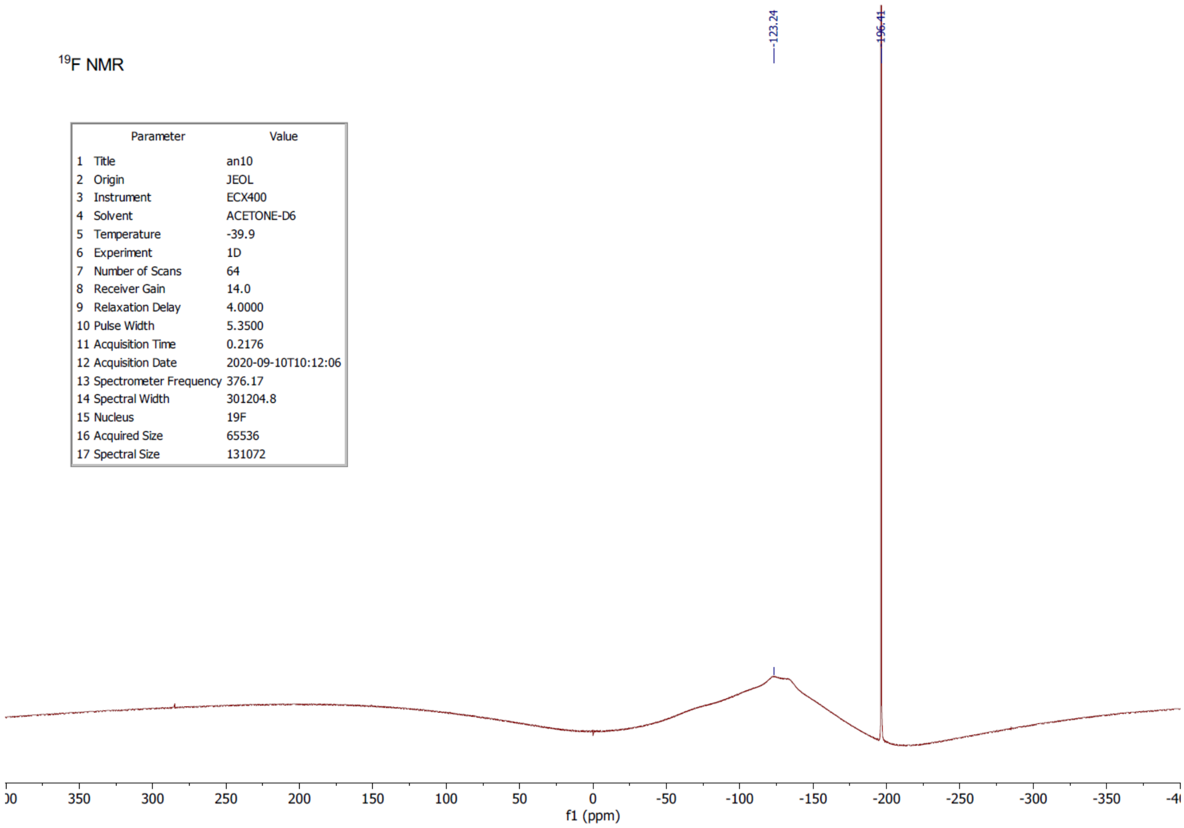

Figure S6. 376 MHz ^{19}F Spectrum of $[C_3H_5O_3]^+[SbF_6]^-$ (**1**).
